# Value-based simulation in healthcare: a new model for metrics reporting

**DOI:** 10.1186/s41077-025-00368-w

**Published:** 2025-07-28

**Authors:** Lisa T. Barker, Michael Meguerdichian, Katie Walker, Sarah Janssens, Rebecca A. Szabo, Connie Lopez, Jared W. Henricksen, Ben Symon

**Affiliations:** 1 Jump Simulation, OSF HealthCare and University of Illinois College of Medicine at Peoria Collaboration, Peoria, IL USA; 2 Simulation Center, NYC Health + Hospitals, New York, NYC USA; 3 Harlem Emergency Department, New York, USA; 4 Mater Misericordiae Limited, Mater Health Simulation Service, Mater Hospitals, Level 4, Duncombe Building, South Brisbane, QLD 4101 Australia; 5 Bond University, Gold Coast, Australia; 6 Mater Mothers Hospital, Brisbane, Australia; 7 Department of Obstetrics, Gynaecology and Newborn Health, Department of Medical Education, University of Melbourne, Melbourne, Australia; 8 The Royal Women’s Hospital, Parkville, VIC Australia; 9 Women’s Health Education, Simulation & Gandel Simulation Service, Parkville, VIC Australia; 10 The Permanente Medical Group, Risk and Patient Safety, Kaiser Permanente, The Permanente Medical Group, Pleasanton, CA USA; 11 University of Utah Health, Salt Lake City, USA; 12 School of Clinical Medicine, University of Queensland, Brisbane, Australia

**Keywords:** Value-based simulation, Healthcare simulation, Program evaluation, Organizational impact, Simulation metrics, Translational simulation

## Abstract

**Background:**

Healthcare simulation services are increasingly expected to demonstrate their value—a term that remains highly context-dependent and frequently misunderstood. While traditional models such as Kirkpatrick and Phillips have supported early evaluation efforts, they embed hierarchical assumptions about which types of data matter most. These assumptions can constrain recognition of simulation’s broader contributions and lead to misguided or inefficient measurement practices.

**Main body:**

In this paper, we propose the value-based simulation in healthcare (VBSH) model, an adaptation of Phillips’ framework that offers simulation-specific nomenclature and a service-level lens. Structured as a taxonomy rather than a hierarchy, the VBSH model comprises six freestanding but interdependent categories: Service Products, Program Perceptions, Acquired Expertise, Workplace Performance, System Benefit, and Value Analyses. This model is designed to support simulation teams and organizational leaders in selecting relevant measurement strategies, aligning simulation work with institutional goals, and co-creating metrics that are operationally meaningful.

**Conclusion:**

By reframing simulation as a vector for insight, improvement, and transformation—not just training delivery—the VBSH model aims to shift the conversation from metric power to metric relevance, fostering a more accurate, efficient, and context-aware narrative of simulation’s value in healthcare.

## Introduction

Funding for simulation is often paired with the expectation that teams demonstrate their value. Demonstrating value can be challenging; the concept of “value” is itself subjective—and can be seen very differently by those working in simulation compared to those being asked to pay the bill. Negotiating these different viewpoints is essential. Measuring a program’s value without agreement upon what is valuable to measure can lead to meaningless or misleading data. Like selecting a diagnostic test in clinical practice, we must consider together what to measure, why we are measuring it, and how that data speaks to programmatic value.

Seeking robust models of measurement, services may be drawn to project-based approaches such as Phillips’ Return on Investment model [1]. We argue that Phillips’ model needs adaptation to describe the breadth of value simulation brings to healthcare, and to fundamentally change our conversations with organizational leaders. We propose the value-based simulation in healthcare (VBSH) model. The model is an adaptation of Phillips’ that is simulation specific, connects value to context, and is intentionally a taxonomy rather than a hierarchy. Within this article, we define six VBSH categories, provide examples of measurement, and explain how the simulation community can utilize these categories to report value.

## Background

Our discussion of value-based simulation exists within the paradigm of value-based healthcare. Value-based healthcare considers patient-related outcomes relative to their associated costs [2], placing the patient at the forefront of the value proposition. Simulation should similarly define and measure its value [3–7]. Some simulation-specific guidance has been published regarding analyses of costs [4], benefits of alternatives, and intangible benefits attributable to simulation services; however, our author group maintains that further work is needed.

Pragmatically, improving our approach to measurement is necessary for a simulation service’s long term survival [8]. From a stakeholder’s perspective, providing operational support is fundamentally a business decision based on perceived value to the organization. Failing to connect simulation portfolios to institutional objectives puts services at risk when budgets tighten. A shared language and simulation-specific framework can support simulation services to synchronize their work to institutional priorities.

Historically, there have been a variety of approaches to programmatic evaluation [9–13]. The Kirkpatrick model evaluates the effectiveness of training programs. It evaluates across four levels: participant reactions, learning outcomes, behavioral changes, and organizational results [7]. Phillips’ Return on Investment (ROI) Model [1] adds a fifth level, Return on Investment (ROI). While there are multiple published models, we have adapted Phillips’ ROI model because it combines the training focus of Kirkpatrick [7] with the patient and systems focus emphasized by translational science [14] (Table 1).

**Table 1:**
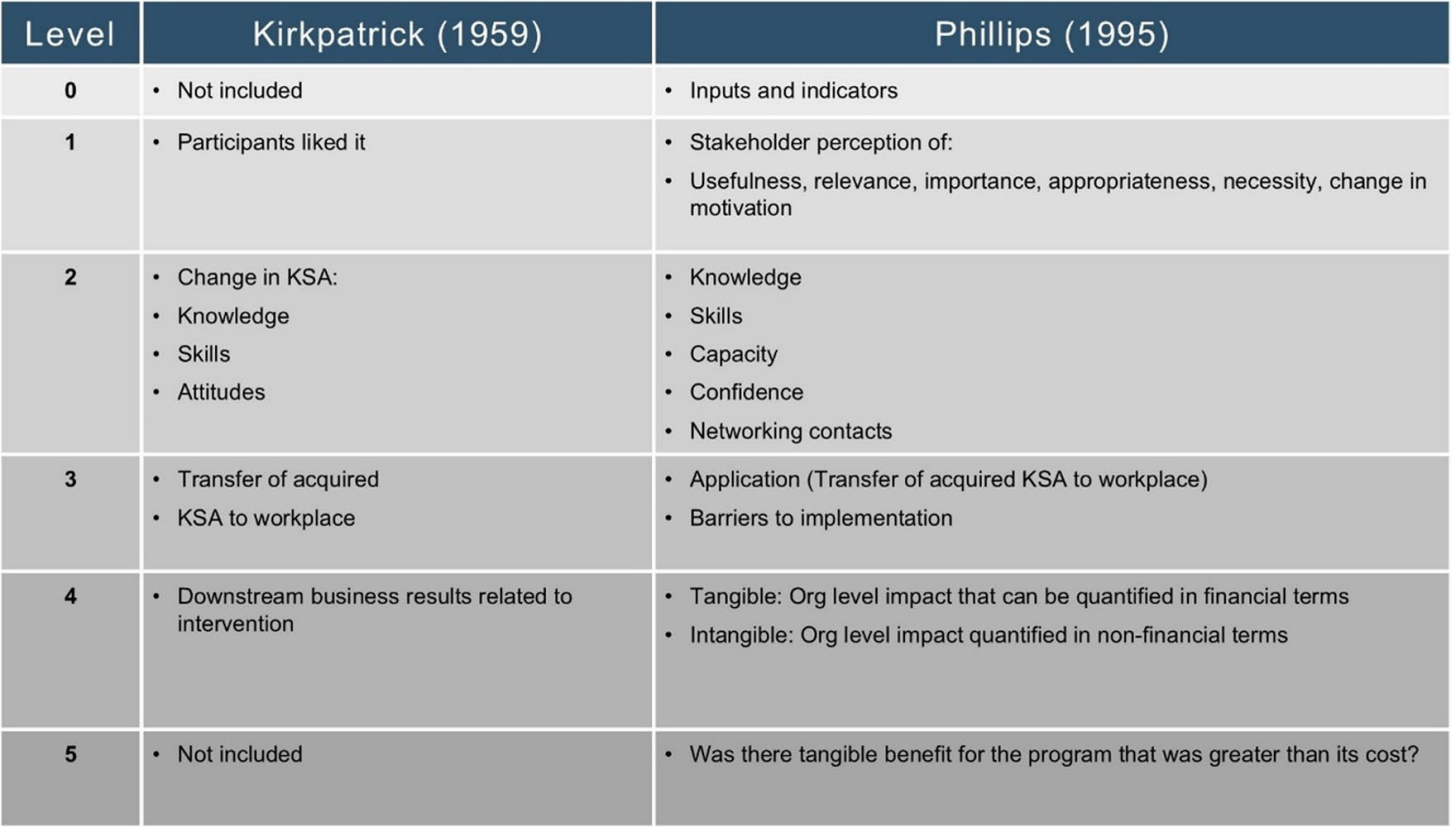
A comparison of Kirkpatrick and Phillips

We believe the value proposition of healthcare simulation can be improved by moving the conversation away from metric *power*, and towards metric *relevance*. The pyramid structure of Phillips’ embeds a hierarchical approach to value that is not always helpful [15]. By displaying different data sets at higher levels in its pyramid, Philips’ implies that some measurement metrics are inherently superior to others. This can lead to organizations dismissing useful data, or consuming excessive resources straining to collect data that directly correlates with clinical outcomes. We argue that there are organizational benefits within every measurement category, and their value depends on the viewpoints and context [3] of the parties involved.

Additionally, simulation is now used more broadly than for education. Many simulation-based interventions (SBI) improve healthcare in other ways, including transmitting culture [16], diagnosing latent safety threats [17, 18], recreating events for root cause analyses [17, 19], and testing new environments [20]. These outcomes are not always directly traceable to patient outcomes or return on investment, but they clearly have value.

A framework for measuring the value of simulation should reflect the breadth of what value simulation can bring. It should have a service-level perspective. It should help organizational leaders understand simulation’s intricacies and help simulation leaders measure or describe their impact more meaningfully. All interested parties benefit from a broader conversation around what simulation can do, what is feasible to measure, and how simulation best helps an organization.

The remainder of our manuscript introduces our adaptation of Philips ROI Model: the value-based simulation in healthcare model (VBSH). The VBSH model is designed to report on all healthcare simulation products and is designed to help organizational and simulation leaders explore simulation’s value.

## The value-based simulation in healthcare (VBSH) model

**Fig. 1:**
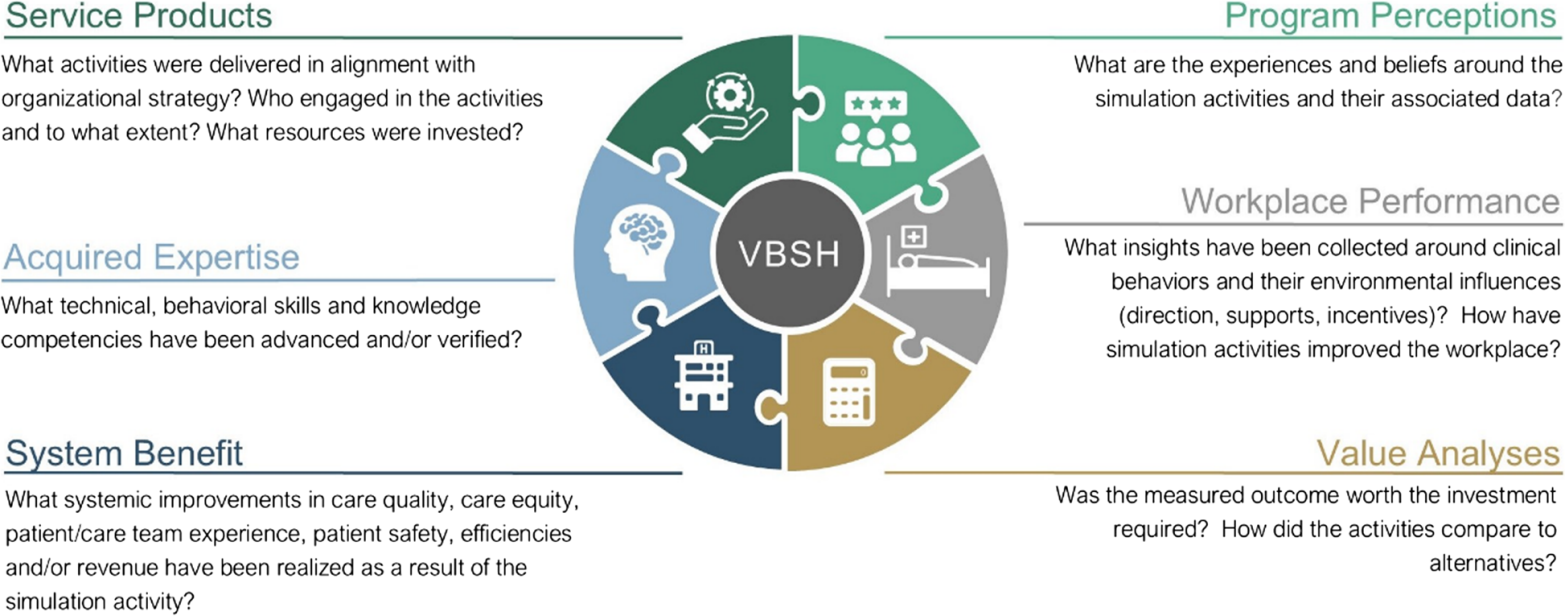
The value-based simulation in healthcare (VBSH) model—graphical abstract

The “value-based simulation in healthcare” (VBSH) Model is a framework for reporting metrics. It captures the benefits a service can provide within six data categories that may be freestanding or interdependent: *Service Products, Program Perceptions, Acquired Expertise, Workplace Performance, System Benefit,* and *Value Analyses* (see Fig. 1).

The six VBSH categories describe all of simulation’s contributions. Categories are named in ways that highlight their service-level impact, nudging simulation teams to consider their work from an organizational perspective. The model incorporates concepts from institutional priorities, medical education, research, and quality improvement in the workplace. Each category contains multiple ways to show simulation’s value, making the value proposition for each program explicit.

The VBSH model is a pie-chart, and categories are not numbered in order to avoid bias that one category is inherently superior to another. Rather than attempting to “reach the top of the pyramid” in pursuit of statistical validity, programs must select which categories are meaningful to report on within their context.

The VBSH model emphasizes that categories can be independent and delineates the conclusions that can be drawn from each one. For example, while Phillips’ includes subjective perceptions of confidence and commitment within their “Learning” level, self-assessment is a limited measure of clinical competence [21–23]. The VBSH Model keeps reactions for an SBI within the category of *Program Perceptions*, maintaining that perceptions are valuable data, but do not prove learning has occurred*.* This is distinct from *Acquired Expertise,* which collates verifiable data that measures evidence of learning and skill acquisition [24].

Within the VBSH model, we have mainly kept financial data within two categories: the *Value Analysis* and *System Benefits* categories. While financial implications are important to many discussions regarding organizational value, they will not always be the most appropriate focus. In our conversations with healthcare leadership, we meet executives with a deeply holistic view of their organizations. They often place high importance on staff and patient experience, issues such as equity and environmental responsibility, and simulation’s ability to help their organization adapt to sudden changes [25]. While financial considerations remain vital to conversations around value, we argue they should be neither omnipotent nor omnipresent. When there is a need to examine outcomes relative to financial cost, all 5 other categories can be incorporated into *Value Analyses*.

We will now define each VBSH category, describe them, explain their context within existing simulation literature, and how each demonstrates value. We provide example measures and case examples from the authors’ experiences in international simulation programs. In our practical implications section, we describe how the model can be implemented into organizational practice.

### VBSH category: service products

The *Service Products* category captures the operational inputs and outputs for all activities of the simulation service. This category provides practical data on the “what”, “who”, and “how” of the service.

Inputs are resources invested into the simulation service. Inputs for simulation include investments of equipment and supplies, space used per session, faculty and staff, learner time, and facility expenses [26]. Outputs are the services and products delivered by the simulation service as a result of this investment. Outputs for simulation services include the number of courses delivered, participant demographics, types of equipment used, scenarios run, and the method, duration, and frequency of the activity [27].

Measurement of *Service Products* is valuable for organizations. Activity content areas and participant demographics contribute to organizational accreditations and regulatory requirements. Providing required training internally helps the service avoid external registration fees and travel expenses for staff. *Service Products* data can be relatively easy to collect and highly useful when synthesized well (Table 2).

**Table 2:**
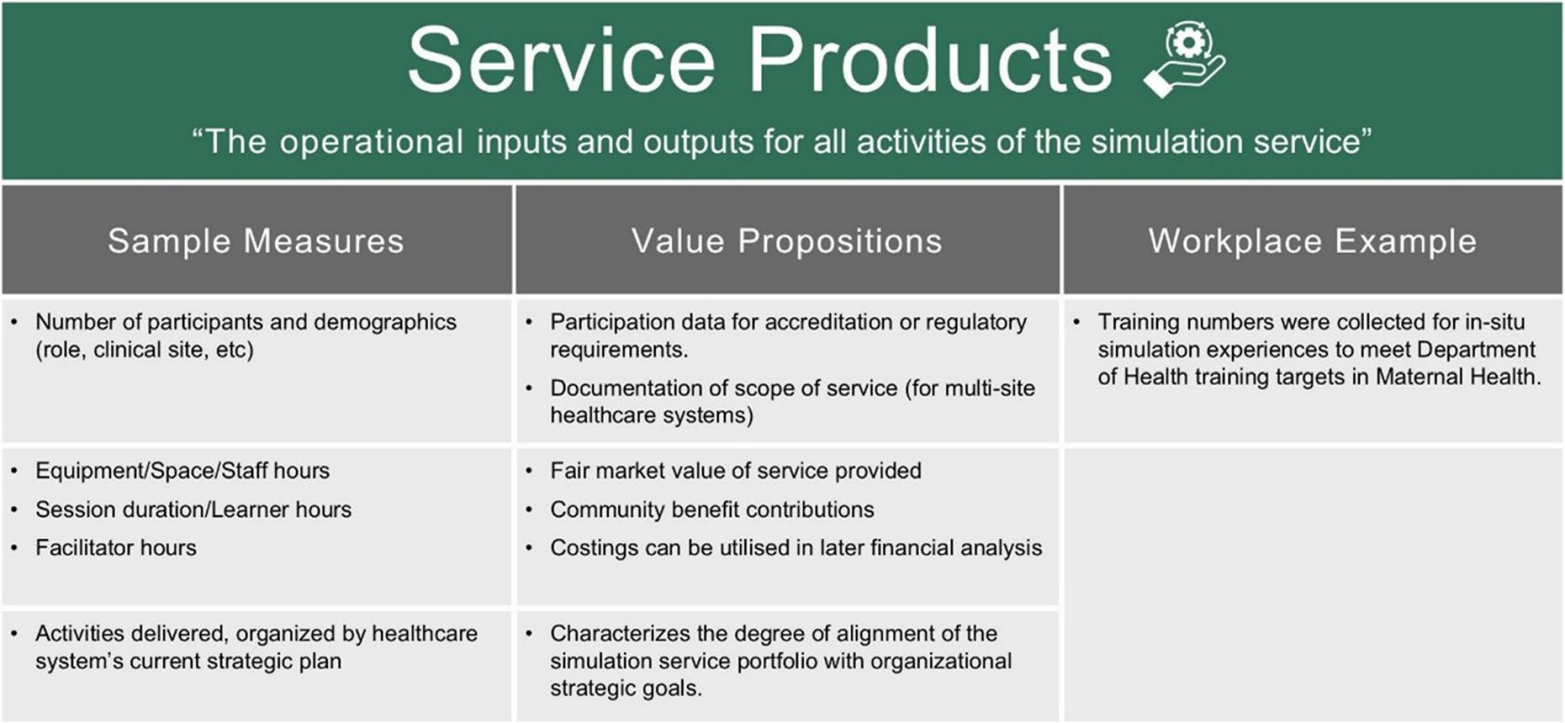
Example measures of service products

#### Case study example

A New York-based simulation service conducted a maternal mortality reduction program with a training target of over 85% of clinical staff. Simulation leaders wanted to prove the clinical impact with maternal mortality metrics, but program sponsors were only interested in the volume of training delivered. Value was efficiently confirmed to sponsors through measurement of *Service Products* by two nurse educators and two data analysts.

### VBSH category: program perceptions

*Program Perceptions* measures how simulation activities are perceived. Traditionally, educators collect participant reactions, but *Program Perceptions* includes feedback from broader aspects of the organization. Staff, supervisors, consumers, and funders likely view simulation activities in different ways, and those perceptions are valuable.

Survey feedback can be easily dismissed, but context is key. For departments implementing new medical techniques or procedures, confidence levels are valuable. Increased self-efficacy predicts behavioral change [28–30]; a confident clinician is more likely to use a new process. For program directors, surveys of clinical leaders detect if there is top-down support for their initiative. Negative perceptions are crucial early warning signals, and positive scores may predict support for future staff attendance. The value of *Program Perceptions* lies in their relevance to what questions are being asked.

In addition to feedback about educational courses and programs, *Program Perceptions* includes feedback generated when using simulation for system improvement. Measurement may include usability and feasibility feedback surveys from adoption frameworks [20]. Staff perceptions of the process being tested inform our understanding of both the process itself and its eventual implementation. Importantly, validated measurement tools during translational projects would not sit under *Program Perceptions*.

*Program Perceptions* are subjective, but subjective data can be highly valuable.

Example measures are shared in Table 3.

**Table 3:**
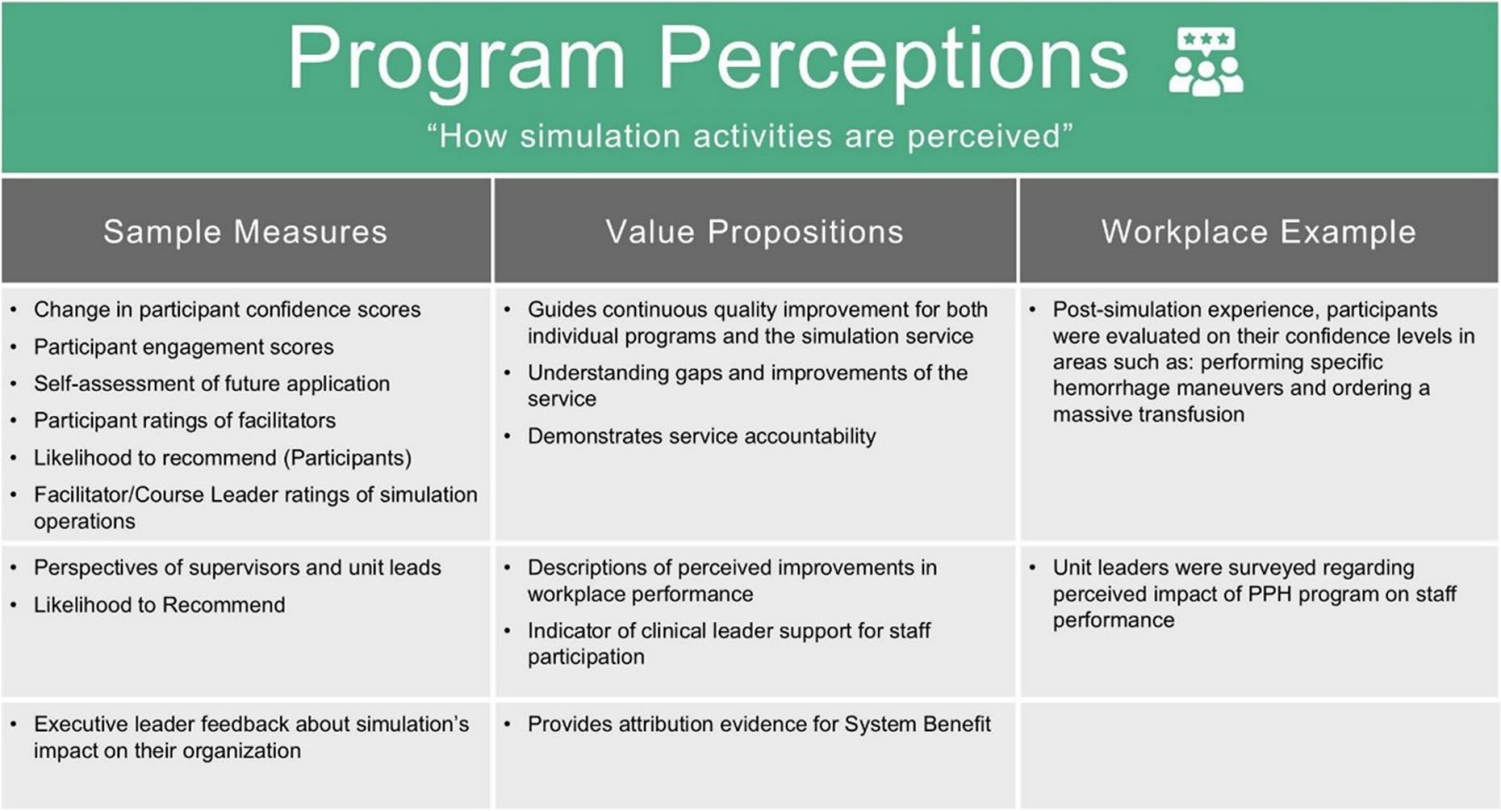
Example measures of program perceptions

*PPH* post-partum hemorrhage

#### Case study example

During review of a simulation service, executive leaders, academic collaborators, and internal simulation staff were sent an electronic survey. Using 5-point Likert scales, survey participants rated how well the service met each Society for Simulation in Healthcare Accreditation Standard. Standards that the service scored less on were then prioritized for quality improvement.

*Program Perceptions* provided value by helping the service to calibrate their performance and respond to their weaknesses.

### VBSH category: acquired expertise

*Acquired Expertise* captures the verified abilities and knowledge gained through simulation-based training. Healthcare simulation has well-established benefits for the development of clinical competence [31–35]. Acquiring expertise means increasing one’s competency in knowledge, technical and behavioral skills as an individual or a team. We have kept the term “expertise” deliberately broad to ensure services can consider this in the context of their own service.

This category within the VBSH model is distinguished from other evaluation models because it excludes subjective self-reports of skill acquisition—that data would fall into *Program Perceptions*. Outcomes reported in this category must be based on defensible assessments using organizationally accepted tools, ideally with validity evidence [29, 36, 37] (Table 4).

**Table 4:**
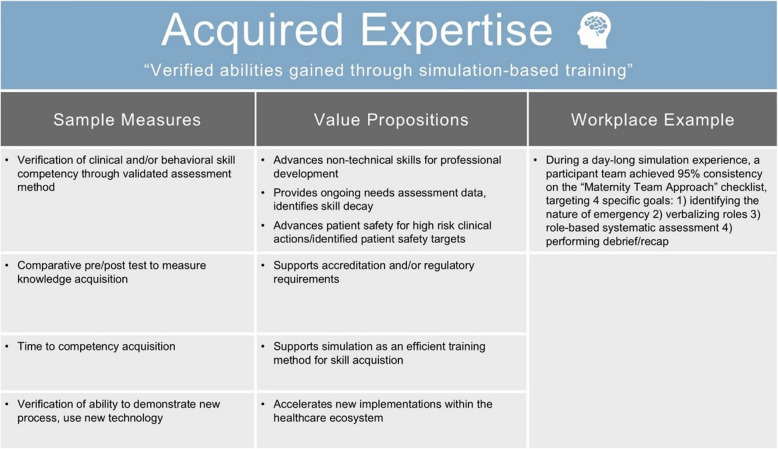
Example measures of acquired expertise

#### Case study example

An Australian service revised its simulation curriculum for maternity emergencies, focusing on behavioral skills training using Visually Enhanced Mental Simulations [38] and Rapid Cycle Deliberate Practice [39]. These techniques were used to teach a standardized approach to critical events in maternity. To verify learning from the new curriculum, the Clinical Teamwork Scale [40] was used to score video of simulation participant teams before and after the curriculum change. Teamwork scores significantly improved. *Acquired Expertise* demonstrated value by confirming team learning had occurred in the new format.

### VBSH category: workplace performance

*Workplace Performance* captures data about the impact of simulation on the workplace, and insights generated by simulation about the workplace itself.

While there is some inter-dependence between *Acquired expertise* and *Workplace performance*, we maintain these two categories warrant separation. *Acquired expertise* captures verified data that measures knowledge and skill acquisition. *Workplace Performance* captures implementation of that learning into clinical environments. We argue these are two separate components of healthcare improvement, and that they often correlate less than anticipated.

Simulation has value in helping decision makers understand complex adaptive systems. A variety of structures exist within the literature to inform this approach, including Human Factors [41] (47) and Systems Engineering Initiative for Patient Safety 2.0 [42]. We have chosen to emphasize translational simulation given translational simulation’s emphasis on considering the “purpose”, “process”, and “conceptual foundations” in a way that can incorporate any relevant theory or framework for system improvement [43]. We acknowledge international variation of terms and methodologies to describe simulation for systems improvement, and like our stance on value, argue organizations must choose the methodology most relevant and useful for their approach.

Within the context of translational simulation, *Workplace Performance* also includes learnings about the organization gathered through simulation. While educational simulation emphasized utilizing simulation and debriefing to shape workplace behavior, more recent methods highlight that data from debriefs and in situ simulations (both educational or translational) can inform our understanding of the workplace itself [44]. For example, Guise et al. categorized changes identified through their mobile in situ work for maternal health [45], and in situ simulations can provide value by identifying opportunities to improve patient safety [46, 47].

Debriefs can also provide deeper insights regarding the cultural and interpersonal barriers hampering workplace initiatives [48]. This data can be used by simulation teams to refine their own approaches or integrate with other quality improvement systems. Simulation’s ability to learn about the “work as done” is a unique value proposition for simulation in healthcare [46, 49–51].

Example measures are included in Table 5.

**Table 5:**
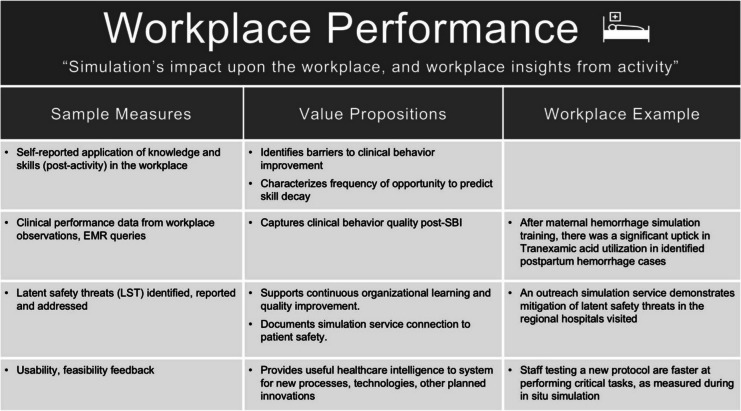
Example measures of Workplace Performance

#### Case study example

A large USA-based healthcare system established a simulation-based patient safety program focused on maternal child health. The program combined simulation-based education with comprehensive workplace data collection to identify needs and outcomes over time [52].

There were many documented improvements in *Workplace Performance*. Key outcomes included improved team performance at the bedside for maternal health emergencies, implementation of a standardized operating room checklist for briefing and debriefing, and the accelerated mitigation of latent safety threats.

*Workplace Performance* demonstrated value by measuring simulation’s impact *on* the workplace, and by helping learn *about* the workplace itself.

### VBSH category: system benefit

*System Benefit* captures simulation’s tangible and intangible impacts on the healthcare system. In particular, it considers the “quintuple aim”, which consists of (1) enhancing the experience of care for individuals; (2) improving the health of populations; (3) reducing the per capita cost of health care; (4) improving the work life of clinicians and staff; (5) advancing health equity across communities and populations [53, 54].

*System Benefit* metrics may be intangible [55] or tangible (financial) [56, 57]. Intangible benefits include positive organization-level outcomes that do not have an identified financial unit value. These include improved quality and safety metrics, and organization-level survey scores for patient experience, safety culture, and employee engagement.

Tangible benefits involve either revenue, cost savings, or cost avoidance. When seeking to demonstrate tangible benefit, it is critical for simulation leaders to engage with their institutional finance team.

Importantly not all quality improvement interventions lead to cost savings or avoidance [58, 59]. Healthcare is unique from other industries in that some operational costs are relatively fixed; they do not vary with swings in patient volumes. Cost reductions are not assured in conjunction with improved care quality, and sometimes costs go up with improved care [58]. While intangible, improved quality and patient safety are valuable targets for simulation-based interventions.

Cost avoidance calculations should be conducted separately from clinical quality performance feedback to avoid subverting incentives that might limit care. Attribution of a system benefit to the simulation service does require defensible data collection and analysis.

Because the VBSH categories can be both freestanding or interdependent, there is some overlap between *Workplace Performance* and *System Benefit.* Data about workplace performance, when collected from the workplace itself, would often legitimately fit into both categories. We recommend presenting that data in the most relevant category for the targeted outcomes. When viewed as discrete understandings about the workplace, this information can be presented as *Workplace Performance*. If instead, the findings are being framed from a system-level lens (e.g., this workplace data confirms improvements in patient safety, equity, outcomes), then they could also be categorized as evidence for *System Benefit.*

Some example measures of system benefit are listed in Table 6.

**Table 6:**
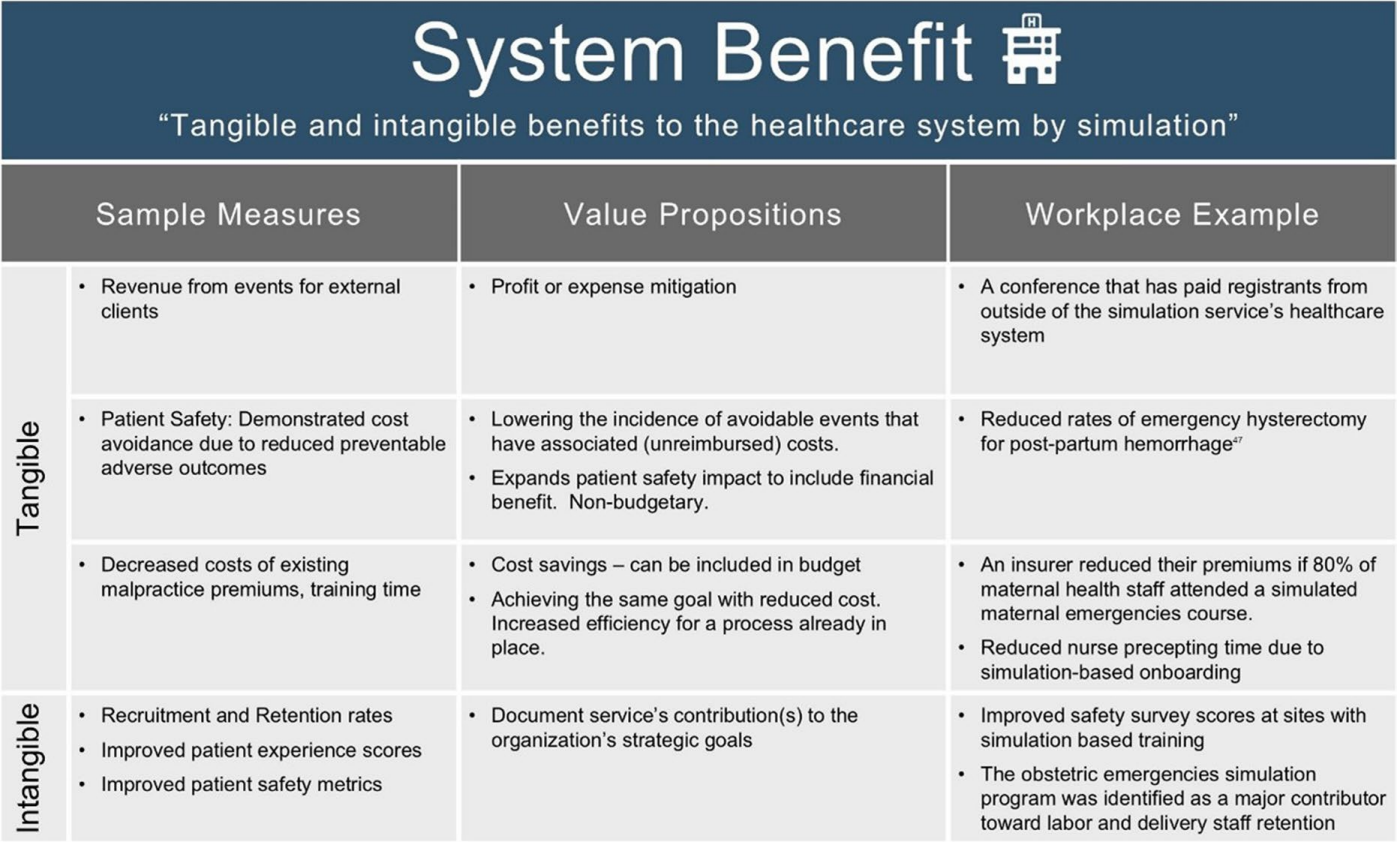
Example measures of system benefit

#### Case study example

An example of tangible *System Benefit* is the “Incentivizing Better Patient Safety” (IBPS) Program. This Australian obstetric harm prevention and reduction program requires participation from 80% of birthing suite staff. If targets are met, the organization receives a partial refund of their medical indemnity premium [60]. The program was described as reducing the risk of incidents in birthing suites and delivered over $AUD five million in premium refunds to health services over 2 years [61].

*System benefits* included improving the health of populations (intangible) and reducing the per capita cost of health care for obstetric patients (tangible).

### VBSH category: value analyses

*Value Analyses* captures simulation-related metrics from the other five categories and reports them in context of their associated financial investments. *Value Analyses* is the category that considers “Was it worth it for the cost?”.

*Value Analyses* are important for Chief Executive Officers (CEOs), Chief Financial Officers (CFOs), and simulation services. Those making funding decisions need accurate information to make the best decisions. For simulation services, incorporating *Value Analyses* supports advocacy for financial sponsorship and aids internal accountability. For example, teams considering expensive equipment purchases, or sending expensive staff to deliver poorly attended courses, may select more financially cautious strategies when costs are incorporated into discussion [62].

This category is entitled *Value Analyses* rather than Return on Investment. Despite increasing focus on a return on investment (ROI) in simulation, the specific approach for cost-related analyses is dependent on measured outcomes and/or a comparator solution [5]. ROI is but one type of analysis that compares the tangible costs and benefits of a single program, but avoids attention to programmatic impact. To mitigate ROI’s focus on finances, some educators have proposed using “return on expectations” (ROE) as an alternative to ROI [63–65]. We argue this approach involves subjective perceptions of value and would fit within *Program Perceptions* category. *Value Analyses* retains a singular focus on data related to financial cost.

*Value Analyses* are important, but a simulation service would be short-sighted to view this category as automatically the most important. *Value Analyses* is one of the available categories to describe the impact of healthcare simulation activities. For simulation services with established operational costs within their organization’s budget, the demonstration of *Acquired Expertise, System Benefit*, or other categories may satisfy organizational leadership, making investment in formal project-based analysis unnecessary.

For example, a program aiming to mitigate burnout may successfully demonstrate its value by connecting to existing target measures such as employee engagement scores. On the other hand, an SBI designed to improve a hospital’s operating room flow may need a cost-effectiveness analysis before deciding to deploy it across the healthcare system.

In summary, formally comparing outcomes in relation to an intervention’s costs is a valuable pursuit for financial accountability in the right circumstances (Table 7).

**Table 7:**
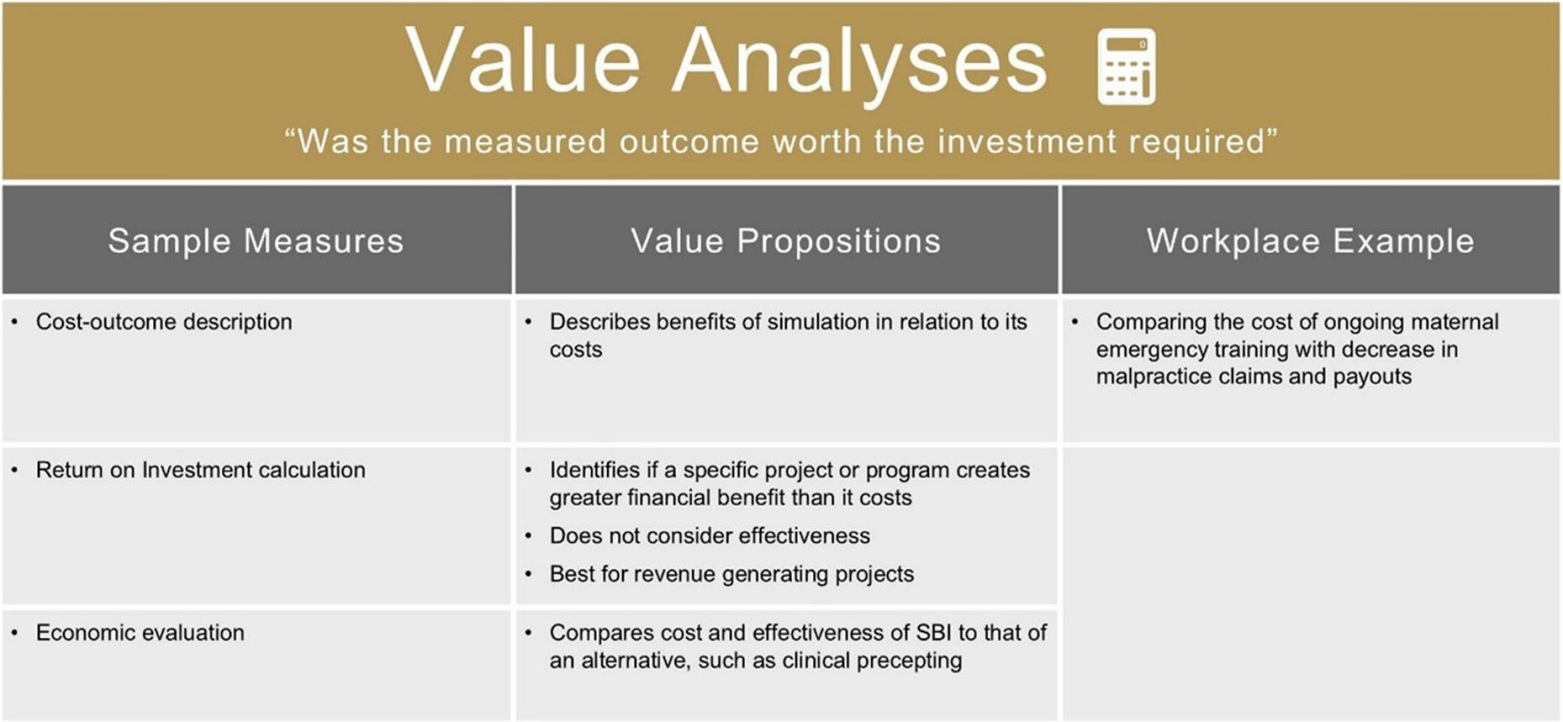
Example measures of value analysis

### Case study example

A US hospital’s shoulder dystocia program was conducted in every labor and delivery unit of a large hospital system with 90% of staff attending at least one session. Claims data was analyzed 3 years after the program was delivered, and for every dollar spent on training, seven dollars in claims costs were avoided for the hospital system. Value to the organization was demonstrated by measuring financial return on investment.

## Discussion

Within our discussion we argue that the VBSH model is an important adaptation of Phillips’. We argue that it promotes a context-specific approach to evaluation, broadens understanding of simulation’s value, and that it refines our approach to measuring value.

### Promoting context-specific evaluation

The VBSH model emphasizes that value is defined collaboratively. Categories are not numbered; they are displayed in a radial, pie chart format to reinforce this stance. The categories have been separated into “slices” within the chart, enabling due consideration of each one. The size of each category slice is visually equal, because different categories will be more or less important for different organizations. Metaphorically speaking, which slice an organization perceives as most desirable will depend on their context and appetite.

### Broadening understanding of simulation’s value

VBSH is designed for use in conversations between organizational leaders and simulation advocates. Each category of the VBSH is named with a service level lens, nudging discussions towards organizational priorities. The six categories help simulation advocates better explain how simulation shapes healthcare ecosystems. Simulation is no longer solely an educational product that is delivered to staff; it is a vector for information and transformation between educators, innovators, clinicians, and leadership. By referencing the model, simulation advocates and executives can more clearly describe or understand the breadth of simulation’s potential.

### Refining our approach to evaluating simulation programs

Each category within the VBSH Model requires a considered, specific approach. The new categories have been described in response to existing gaps in previous approaches to evaluation. *Service Products* captures the “what”, “who”, and “how” of each service and that organizations must consider their inputs and outputs when evaluating programs. *Program Perceptions* broadens whose reactions should be collected, and highlights that subjective data is valuable when applied in the right context. *Acquired Expertise* promotes academic rigor in measuring competency, avoiding the implication that self-ratings accurately reflect mastery. *Workplace Performance* acknowledges translational simulation’s role in transforming healthcare systems, and that the relationship between simulation and the workplace should be bidirectional and iterative. It also reinforces that education cannot always be directly correlated with bedside outcomes. *System Benefit* affirms that value in healthcare is not always reflected by decreased cost. *Value Analyses* ensures that financial implications and analysis remain an essential consideration for value-based healthcare delivery.

## Practical implications

Within the author group, we have utilized the VBSH model to explain what our own simulation services can achieve, and to help us synchronize priorities and initiatives with organizational vision. It allows us to explain what can, and what cannot be concluded from our metrics reporting. It helps us judiciously select the right measurement targets for our endeavors, and helps organizational leaders understand what we can do.

Our drive to publish this framework is influenced by our experiences watching simulation leaders negotiate value in counterproductive ways. We have seen simulation leaders over-promise changes in patient outcome metrics, which can set their service up for failure at performance review when it is found statistically impossible to prove. We have seen organizational leaders with strong enthusiasm for simulation push for educational interventions that have a limited likelihood of generating meaningful impact. We have also seen simulation services waste resources attempting to collect data they think will prove their worth, without considering the opportunity cost of collecting their chosen reporting metrics.

The VBSH model can be shared with executive and simulation teams can make suggestions on where simulation can best contribute to solving a particular workplace problem. To give additional context, Table 8 provides examples of different measurement metrics within each category.

**Table 8:**
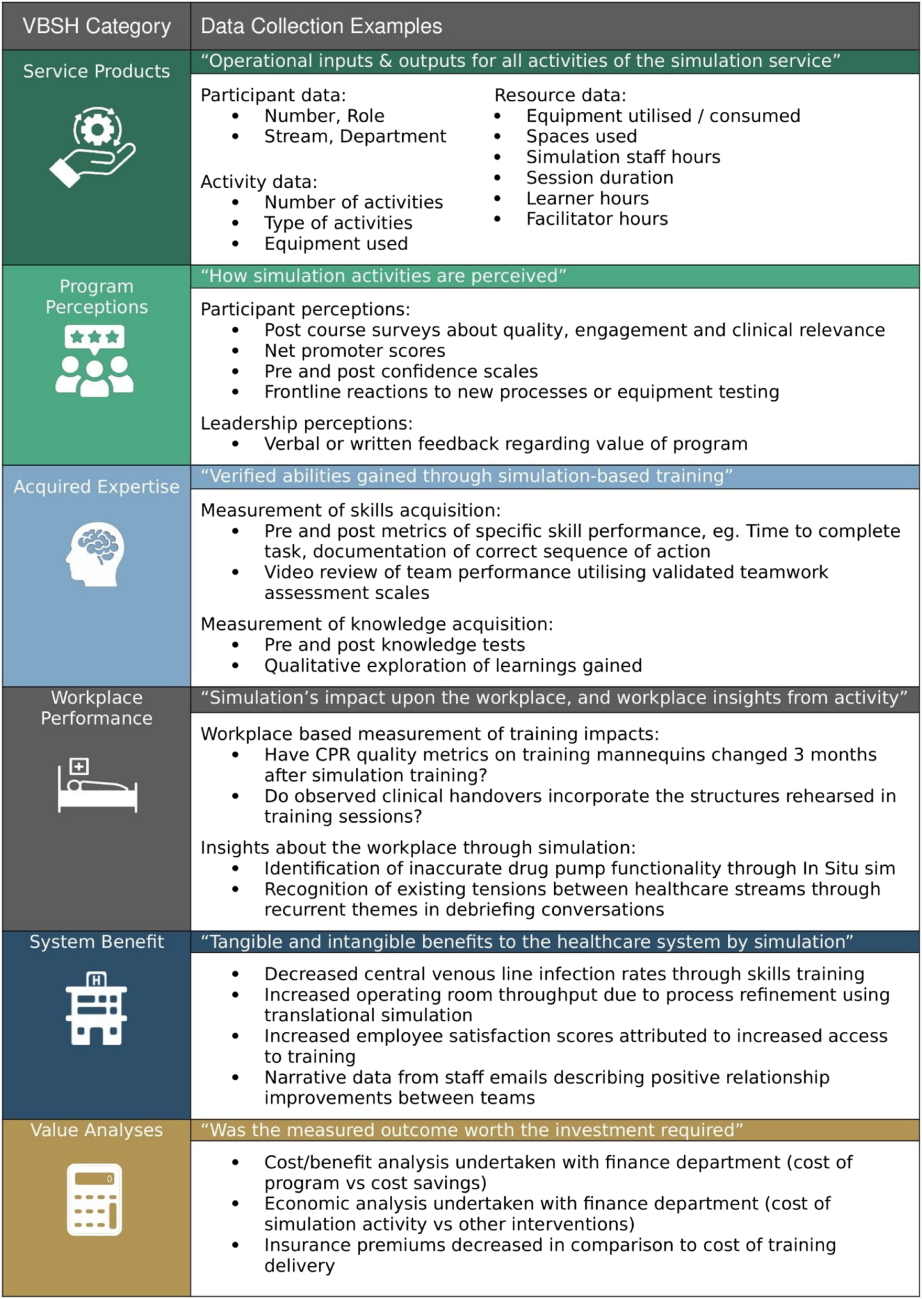
Data collection examples of the six VBSH categories

We hesitate to provide a decision tree or more tools for guiding conversations with leadership and sponsors, as these will be culturally and regionally specific. We have instead provided a fictional case study as an example of how VBSH can be used.

## Case study

A large health service is experiencing higher than usual frequencies of wrong site surgery. Governance believes that compliance with the WHO Surgical Safety Checklist is limited [66], and that operating room nurses hesitate to speak up to surgeons when the checklist is not initiated.

The simulation team is approached for assistance, and executive initially requests additional speaking up for safety training hospital-wide. They ask for an increased volume of education to be delivered to operating room staff, a request for an increase in *Service Products*.

The simulation service argues that education can have a limited impact on workplace culture change. They orient executives to the VBSH model and highlight that:

- To understand barriers to checklist use, translational simulation can be used to understand workplace frustrations and cultural issues preventing engagement (*Workplace Performance*)
- While a decrease in wrong site surgery and subsequent legal costs is desirable, the frequency is already rare, and a financial return on investment may not be statistically demonstrable with current patient volume. (*Value Analyses)*

Using Table 8 together, executive and simulation leaders decide that the most valuable metrics for their context are likely:

- ⚬ *Workplace Performance*: using qualitative data from debriefs to understand barriers to engagement in the operating room
- ⚬ *Program Perceptions*: staff feedback on checklist prototypes tested in the sim lab
- ⚬ *System Benefit*: improvement in checklist integration and workplace psychological safety, as demonstrated by workshop feedback from operating room staff

The simulation service adapts its strategy to prioritize these strategies and to collect the relevant metrics.

## Limitations

The VBSH model and the categories provided are informed by the authors’ experiences in simulation services worldwide and existing literature. It has not yet been formally tested in a healthcare organization in its entirety, nor intended to be a full economic evaluation model. There are many approaches to calculating tangible value, and analyses are best conducted with local finance experts. Simulation is an adaptive methodology, and future uses may be identified that do not fit within the current model.

Like other approaches to measurement, implementation will be influenced by data curation challenges, culture, available resources, and leadership support. Barriers may be overcome by integration with pre-existing data collection systems, positive relationships with change agents, and identifying hospital movements with symbiotic goals. We acknowledge a Western bias to our perspective, given all authors come from English-speaking countries within the Global North.

As an author group, we acknowledge Kirkpatrick and Phillip’s models are foundational to this approach and acknowledge their influence. We hope for its future integration in the workplace and that it helps with future conversations in simulation labs and C-suites alike.

## Future directions

We anticipate future use of the VBSH model in case presentations and academic publication, and hope that an ongoing conversation with the simulation community will aid us in clarifying, refining, or adapting the model further. We acknowledge we stand on the shoulders of giants in the field and look forward to future innovations from others to continue advancing our understanding of value-based simulation in healthcare.

## Conclusion

Current models constrain our language, approach, and understanding of simulation’s value. The VBSH model fills this gap by providing us with better nomenclature, centralizing co-creation of metrics to achieve organizational goals, facilitating reporting of all simulation experiences beyond training, and broadening our own understanding of what “value” is and how it should be measured. Simulation in healthcare will continue to thrive if we can demonstrate value effectively.

The six categories (*Service Products, Program Perceptions, Acquired Expertise, Workplace Performance, System Benefit,* and *Value Analyses*) are intentionally freed from hierarchy and aid services in building a narrative around their contributions. The emphasis on co-creation means sponsors and services together can build an approach that is targeted, achievable, and efficient. In doing so, we reframe the way we see “value” within healthcare organizations, enabling a narrative about simulation that is more accurate and operationally meaningful.

### List of terms

Organizational learning: the process of an organization creating, retaining, and transferring knowledge, leading to continuous improvement and adaptation. (Oxford Reference)

Translational simulation: describes healthcare simulation focused directly on improving patient care and healthcare systems, through diagnosing safety and performance issues and delivering simulation-based intervention, irrespective of the location, modality, or content of the simulation (Brazil, V., 2017).

## Data Availability

No datasets were generated or analysed during the current study.

## Abbreviations

VBSH: Value-based simulation in healthcare
VEMS: Visually enhanced mental simulations
SBE: Simulation-based education
SBI: Simulation-based interventions
RCDP: Rapid-cycle deliberate practice
ROI: Return on investment
ROE: Return on expectations
